# Heat shock protein 72 suppresses apoptosis by increasing the stability of X-linked inhibitor of apoptosis protein in renal ischemia/reperfusion injury

**DOI:** 10.3892/mmr.2014.2939

**Published:** 2014-11-13

**Authors:** BAIYU ZHANG, RONG RONG, HUIYAN LI, XUAN PENG, LIPING XIONG, YIHAN WANG, XUEQING YU, HAIPING MAO

**Affiliations:** 1 Department of Nephrology, The First Affiliated Hospital, Sun Yat-sen University, Key Laboratory of Nephrology, Ministry of Health of China, Guangzhou, Guangdong 510080, P.R. China; 2 Department of Rehabilitation, The Second Affiliated Hospital, Sun Yat-sen University, Guangzhou, Guangdong 510120, P.R. China; 3 Laboratory for Kidney Pathology, Inc., Nashville, TN 37203, USA

**Keywords:** heat shock protein 72, apoptosis, acute kidney injury, X-linked inhibitor of apoptosis protein, second mitochondria-derived activator of caspases/direct IAP-binding protein with low PI

## Abstract

X-linked inhibitor of apoptosis protein (XIAP) negatively regulates apoptotic pathways at a post-mitochondrial level. XIAP functions by directly binding and inhibiting activation of specific caspases. Upon apoptotic stimuli, mitochondrial second mitochondria-derived activator of caspases (Smac)/direct IAP-binding protein with low PI (DIABLO) is released into the cytosol, which results in displacement of XIAP from caspases. Heat shock protein 72 (HSP72), an anti-apoptotic protein, prevents mitochondrial injury resulting from acute renal ischemia/reperfusion (I/R), its role in Smac/DIABLO and XIAP signaling remains to be elucidated. In the present study, the hypothesis that HSP72 prevents XIAP degradation *in vivo* and *in vitro* was assessed. To this purpose, a rat model of I/R injury was used to investigate the renoprotective role of HSP72 by treatment with geranylgeranylacetone (GGA), a specific inducer of HSP72. The mechanism of the cytoprotective properties of HSP72 was also investigated *in vitro* using adenovirus-mediated overexpression of HSP72 in adenosine triphosphate (ATP)-depleted human kidney 2 (HK-2) cells. Pre-conditioning rats with GGA attenuated renal tubular cell damage, reduced cell apoptosis, preserved XIAP protein content and improved renal function following I/R injury. An *in vitro* study was performed in which cells were transiently exposed to 5 mM sodium cyanide in a glucose-free medium in order to induce apoptosis. Compared with the control, overexpression of HSP72 inhibited Smac/DIABLO release from the mitochondria and increased levels of XIAP and pro-caspase 3 in ATP-depleted HK-2 cells. In addition, HSP72 interacted with Smac/DIABLO. The present data demonstrates that HSP72 preserves renal function in I/R injury through its anti-apoptotic effects, which act by suppressing mitochondrial Smac/DIABLO release and preserving XIAP protein content.

## Introduction

Apoptosis is the major pathogenetic mechanism of early tubular cell death in ischemia/reperfusion (I/R)-induced acute kidney injury (AKI). Under I/R conditions, disruption of the outer mitochondrial membrane potential and permeability, leading to the release of multiple toxic proteins, has been implicated in activating apoptotic signaling (1,2). However, the cell death pathway exists in a state of dynamic equilibrium between pro and anti-apoptotic effector molecules. The inhibitor of apoptosis proteins (IAP), for example, negatively regulate apoptotic signaling at a post-mitochondrial level. Among the IAP family, X-linked inhibitor of apoptosis protein (XIAP) appears to be the most potent caspase inhibitor by directly binding and inhibiting caspase targets, including caspase-3, 7 and 9. In addition, in response to apoptotic stimuli, the inhibitory function of XIAP can be antagonized by second mitochondria-derived activator of caspases (Smac)/direct IAP-binding protein with low PI (DIABLO) (3–7), which is also released from mitochondria as cytochrome c. In addition, interaction of XIAP with Smac/DIABLO has been demonstrated to mediate apoptosis following diverse insults, including ischemia (8–10), oxidative stress (11) and ultraviolet radiation (12,13). Despite these findings, the role of Smac/DIABLO and XIAP in renal I/R remains to be elucidated.

Heat shock protein 72 (HSP72), a major stress inducible protein, functions as a molecular chaperone in protein folding, transport and degradation. Previous studies from our laboratory (Renal Section, Department of Medicine, Boston Medical Center, Boston University, Boston, MA, USA) and elsewhere have revealed that HSP72 protects renal epithelial cells from apoptosis by reducing mitochondrial membrane injury and inhibiting mitochondrial release of cytochrome c and apoptosis-inducing factor (AIF) (7,14,15). Furthermore, this evidence also suggests that HSP72 attenuates renal fibrosis through inhibiting epithelial-to-mesenchymal transition (16,17). Thus, induction of HSP72 may have wide-ranging effects in the treatment of acute and chronic renal injury. However, it remains to be elucidated whether HSP72 protects against I/R-induced renal tubular cell injury through modulation of Smac/DIABLO and XIAP signaling.

In the present study, it was hypothesized that HSP72 reduces mitochondrial Smac/DIABLO release, prevents XIAP degradation and thereby promotes tubular cell survival in renal I/R injury.

## Materials and methods

### Reagents and antibodies

Geranylgeranylacetone (GGA) was obtained from Eisai China (Shanghai, China). Terminal deoxynucleotidyl transferase-mediated dUTP nick end labeling (TUNEL) assay kits (fluorescent), annexin V fluorescein isothiocyanate apoptosis detection kits and protease inhibitors were obtained from Calbiochem (San Diego, CA, USA). In addition, the following antibodies were used: mouse anti-human HSP72 (1:1,000; Stressgen Biotechnologies, Victoria, BC, Canada), rabbit anti-human XIAP (1:1,000; BD Biosciences, San Jose, CA, USA), mouse anti-human Smac/DIABLO (1:1,000; BD Biosciences), rabbit anti human pro caspase 3 (1:500; Santa Cruz Biotechnology, Inc., Santa Cruz, CA, USA) and mouse anti-human β-actin (1:2,000; Boster, Wuhan, China). Horseradish peroxidase-conjugated anti-mouse IgG and horseradish peroxidase conjugated anti-rabbit IgG were obtained from Jackson ImmunoResearch (West Grove, PA, USA). All remaining reagents were purchased from Sigma-Aldrich (St. Louis, MO, USA).

### Cell culture and treatment

An immortalized proximal tubule epithelial cell line from normal adult human kidney (HK-2) was purchased from the American Type Culture Collection (Rockville, MD, USA). Cells were cultured at 37°C in a 5% carbon dioxide atmosphere in Dulbecco’s modified Eagle’s medium mixed 1:1 (vol:vol) with F12 medium (Invitrogen Life Technologies, Carlsbad, CA, USA) supplemented with 10% fetal bovine serum. Cells were grown to 70–80% confluence and subjected to serum-deprivation for 24 h prior to experimental manipulation.

### Induction of HSP72

HSP72 protein content was enhanced by coinfecting HK-2 cells with adenoviruses containing wild-type human HSP72 and green fluorescent protein (AdvTR5/HSP72-GFP) located on separate cistrons induced by a tetracyclin-regulated promoter (AdvCMV/tTA) as described previously (16). To induce optimal renal HSP72 expression, GGA was emulsified with 5% gum arabic and 0.008% tocopherol and administered to rats as previously described (16). Briefly, rats received daily oral administration with 400 mg/kg GGA, starting one day prior to surgery and continuing throughout I/R or sham surgery. Control animals were administered the same volume of components without GGA (vehicle).

### Animals

The experiments were performed with adult male Sprague-Dawley rats weighing 200–250 g maintained with free access to water and standard food. Renal I/R studies were performed using the protocol approved by the Animal Care and Use Committee of the Sun Yat-sen University (Guangzhou, China). Rats were randomly allocated into three groups: *i*) Sham-surgery controls receiving vehicle (n=8); *ii*) I/R group receiving vehicle (n=8); *iii*) I/R group receiving GGA (n=8).

Renal I/R injury was induced as previously described (18). Briefly, rats were anesthetized by an intraperitoneal injection of chloral hydrate (350 mg/kg) and placed on a sterile disposable towel over a warming pad. A midline incision was made and the renal pedicles were bluntly dissected. The left renal pedicles were occluded with a nontraumatic vascular clamp for 45 min and subsequently allowed to reperfuse following removal of the clamp. The right kidney was removed. The incision was closed with 3-0 silk and rats were returned to cages to recover. The rats in the sham-surgery group were treated in an identical manner, with the exception of clamping of the renal pedicles. Animals were sacrificed following 24 h of reperfusion. Subsequently, blood samples were collected by heart puncture for measurement of creatinine and urea nitrogen and the left kidney was harvested and subjected to further investigation as described below.

### Renal function examination

Blood urea nitrogen (BUN) and creatinine levels were measured using a QuantiChrom BUN or Creatinine assay kit (BioAssay Systems, Hayward, CA, USA).

### Histological analysis and apoptosis assay

Kidney tissues were fixed in 10% phosphate-buffered formalin, embedded in paraffin, sectioned at 3 μm thickness and then stained with hematoxylin and eosin or periodic acid-Schiff (PAS). Histological examinations were performed in a blinded manner and scores were calculated on the basis of the percentage of damaged tubules in 20 randomly selected cortical tubules with visible basement membranes at ×400 magnification (Axioplan 2 imaging; Carl Zeiss, Oberkochen, Germany). A five-point scale was used: 0, Normal; 1, <10%; 2, 11–25%; 3, 25–50%; 4, 51–75% and 5, >75%.

Apoptosis was quantified in histological sections using a commercially available TACS TdT-Fluor *In Situ* Apoptosis Detection kit (R&D Systems, Inc., Minneapolis, MN, USA) according to the manufacturer’s instructions and our previous study (16). Briefly, paraffin-embedded kidney sections were deparaffinized, permeabilized and rehydrated. Slides were incubated with a TUNEL reaction mixture containing terminal deoxynucleotidyl transferase. Positive staining was identified in the cell nucleus with DNA fragmentation under confocal microscopy (Zeiss LSM 510 META; Carl Zeiss) microscopy and expressed as apoptotic cells per high-power field.

### Western blot analysis

Kidney cortex and harvested cultured cells were homogenized in lysis buffer supplemented with a protease inhibitor cocktail (Cell Signaling Technology, Inc., Beverly, MA, USA). Cytosolic protein fractions were obtained through incubation of cells with digitonin buffer [10 mM piperazine-N,N′-bis(2-ethanesulfonic acid), pH 6.8, 0.015% (wt/vol) digitonin, 300 mM sucrose, 100 mM NaCl, 3 mM MgCl_2_, 5 mM EDTA and 1 mM phenylmethylsulfonyl fluoride] for 10 min at 4°C (7). The supernatants of tissue, cell lysates and cytosolic protein extracts were extracted, subjected to protein assay and mixed with sodium dodecyl sulfate (SDS) loading buffer. Samples were loaded and separated by 12% SDS polyacrylamide gels (SDS-PAGE), electrotransferred onto a nitrocellulose membrane, blotted with the designated antibodies and then detected by enhanced chemiluminescence (Amersham Pharmacia Biotech, Amersham, UK). Densitometric quantification was performed with the image analysis program (Fluorchem™ 8900; Alpha Innotech, San Leandro, CA, USA).

### Immunoprecipitation analysis

Cytosolic protein fractions were dissolved in immunoprecipitation buffer (0.5–1 mg of protein/ml) as described previously (17). The cell lysates were incubated overnight at 4°C with a polyclonal rabbit antibody directed against human XIAP (2 μg/mg protein/ml immunoprecipitation buffer; BD Biosciences). The immunocomplexes were isolated by incubation at 4°C with Protein A/G PLUS agarose beads (Pierce Biotechnology Inc., Rockford, IL, USA) for 2 h, washed three times with the immunoprecipitation buffer and analyzed using the indicated antibody by SDS-PAGE and western blotting.

### Statistical analysis

All results are expressed as the mean ± standard error of the mean. Analysis was performed with standard statistical software (SPSS 11.0; SPSS, Inc., Chicago, IL, USA). Comparison among groups was performed using a one-way analysis of variance followed by the Student-Newman-Keuls test. P<0.05 was considered to indicate a statistically significant difference.

## Results

### GGA attenuates I/R-induced renal injury

**Figure 1:**
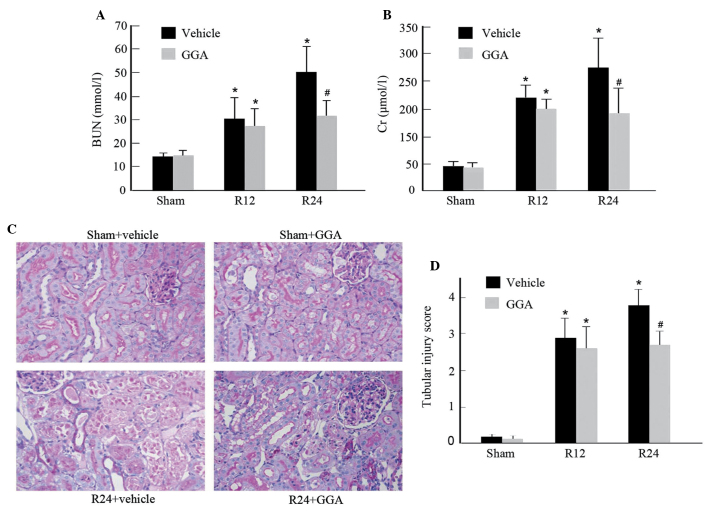
Effects of pretreatment with GGA on ischemia/reperfusion-induced renal injury. (A) BUN levels were examined after 12 h (R12) and 24 h (R24) of renal ischemia/reperfusion. (B) Serum creatinine levels were assessed following surgery (12 and 24 h). (C) Representative micrographs of periodic acid-Schiff stained renal sections from different groups after 24 h of reperfusion (magnification, ×400). (D) Semiquantitative scores of tubular injury in hematoxylin and eosin-stained kidney sections using a scale of 0 to 5 as described in Materials and methods. Values in A, B and D are presented as the mean ± standard error of the mean; ^*^P<0.01 versus sham-surgery rats; ^#^P<0.05 versus vehicle-treated rats. GGA, geranylgeranylacetone; BUN, blood urea nitrogen.

Previous studies from our laboratory and others have demonstrated that orally administered GGA selectively enhances expression of HSP72 in the kidney (16,19). In order to assess the protective roles of GGA in acute kidney injury, renal function was evaluated in a rat I/R injury model. Compared with the sham-surgery group, the I/R rats with vehicle alone exhibited marked and progressive elevation in the levels of BUN and creatinine. By contrast, GGA administration significantly improved renal dysfunction 24 h after reperfusion (Fig. 1A and B). Concordantly, histological analysis of PAS staining revealed that I/R in the vehicle group caused significant brush border loss, detached tubular epithelium, cast formation and tubular dilation compared with the sham-surgery group. However, GGA treatment exhibited significant improvements in renal morphology (Fig. 1C) and reduced the tubular injury score (Fig. 1D). Of note, GGA did not alter renal function and morphology in rats subjected to sham I/R.

### GGA inhibits apoptosis and XIAP degradation

**Figure 2:**
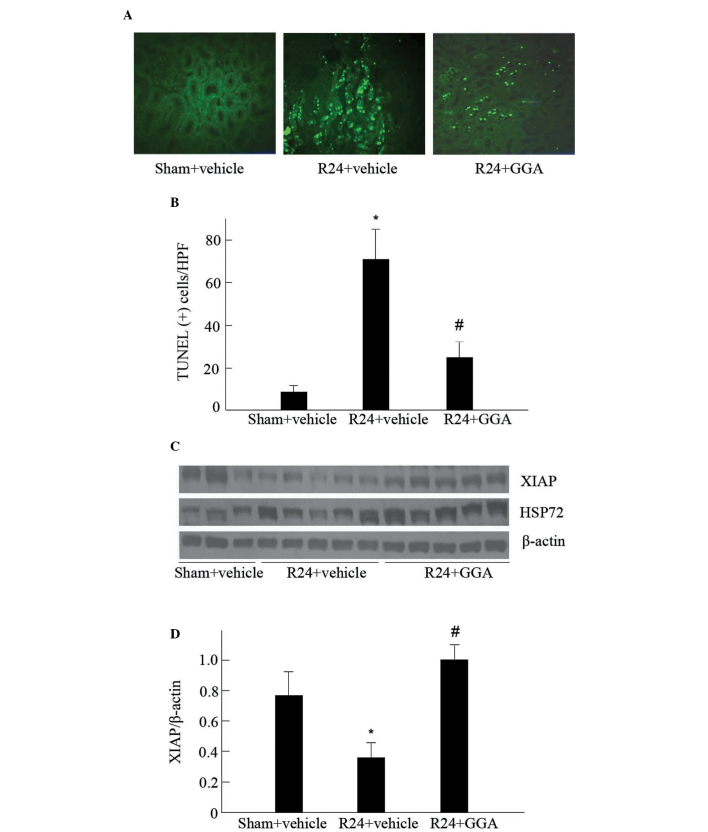
Effects of GGA on renal tubular cell apoptosis. (A) Representative images of TUNEL in different groups as indicated (magnification, ×400). (B) Morphometric analysis of the number of TUNEL-positive cells per high-power field in microscopy. (C) Expression of XIAP and HSP72 in the kidney was examined by immunoblot, β-actin served as a loading control. (D) Graphic representation of XIAP protein levels in different groups, as indicated following normalization with β-actin content. Data in B and D are expressed as the mean ± standard error of the mean; ^*^P<0.01 versus sham-surgery rats; ^#^P<0.05 versus vehicle-treated rats. GGA, geranylgeranylacetone; XIAP, X-linked inhibitor of apoptosis protein; TUNEL, terminal deoxynucleotidyl transferase-mediated dUTP nick end labeling; HSP72, heat shock protein 72.

To examine the potential mechanism for the renal protective effect of GGA, the apoptosis of tubular cells was detected using TUNEL staining. As shown in Fig. 2A, renal TUNEL-positive cells, predominantly located at the proximal tubules, were increased in vehicle-treated I/R kidneys, as compared with those of the sham-surgery group. A significantly lower number of apoptotic cells were observed in the GGA-treated kidneys subjected to I/R (Fig. 2A and B).

XIAP has been observed to suppress cell death by directly inhibiting caspase activity (20). Therefore, the impact of GGA on the steady-state levels of XIAP were examined. As shown in Fig. 2C and D, XIAP protein levels in the kidney were significantly reduced following I/R injury in the vehicle-treated rats, which was consistent with increased apoptosis. The administration of GGA markedly enhanced HSP72 expression, corresponding with preservation of XIAP protein contents. These findings suggest that HSP72 may protect the kidney against I/R-induced injury, at least in part, through inhibiting XIAP degradation.

### HSP72 prevents Smac/DIABLO release and caspase 3 activation

**Figure 3:**
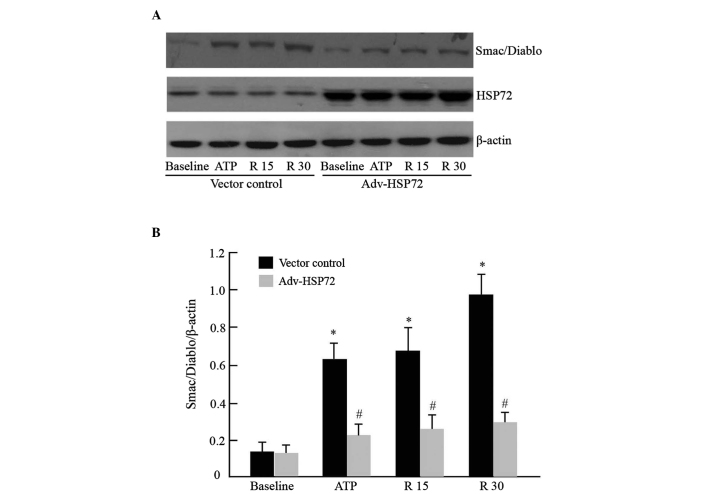
Effects of HSP72 on mitochondrial Smac/DIABLO release. (A) Human kidney-2 cells were infected with adenovirus encoding empty vector or wild type HSP72. Smac/DIABLO leakage into the cytosol was assessed in digitonin-treated cells at baseline, immediately after 1 h of exposure to a metabolic inhibitor (ATP depletion) and after 15 min (R15) or 30 min (R30) of recovery. (B) Densitometric analysis of the effect of HSP72 expression on Smac/DIABLO leakage normalized with β-actin in cells. Data are presented as the mean ± standard error of the mean; n=3 per treatment; ^*^P<0.01 versus empty vector control; ^#^P<0.05 versus metabolic inhibitor-treated cells without HSP72 overexpression. ATP, adenosine triphosphate; HSP72, heat shock protein 72; Smac/DIABLO, second mitochondria-derived activator of caspases/direct IAP-binding protein with low PI.

In response to apoptotic stimuli, Smac/DIABLO protein is redistributed from the mitochondria to the cytosol, binds to XIAP and activates the apoptosome complex. As GGA exposure preserved XIAP protein levels, attenuated apoptosis and protected against I/R renal injury in rats, the function of HSP72 in regulating mitochondrial Smac/DIABLO translocation and caspase 3 activation were examined in cultured epithelial tubular cells subjected to ATP depletion, as previously described (7). Under normal conditions, Smac/DIABLO was not detected in the cytosol of empty vector cells. ATP depletion caused a marked increase in cytosolic Smac/DIABLO. The quantity of Smac/DIABLO progressively enhanced during recovery from transient ATP depletion. However, HSP72 overexpression significantly inhibited mitochondrial Smac/DIABLO release (Fig. 3A and B). Concordantly, levels of pro-caspase 3 were reduced in metabolic inhibitor-treated control cells, whereas caspase 3 activation was efficiently inhibited in HSP72 overexpressing cells (Fig. 3C and D). Without metabolic inhibitor exposure, HSP72 overexpression *per se* did not affect Smac/DIABLO leakage and pro-caspase 3 content. This result suggested that HSP72 may confer protective effects in part by inhibiting mitochondrial Smac/DIABLO protein release.

### HSP72 stabilizes XIAP protein

**Figure 4:**
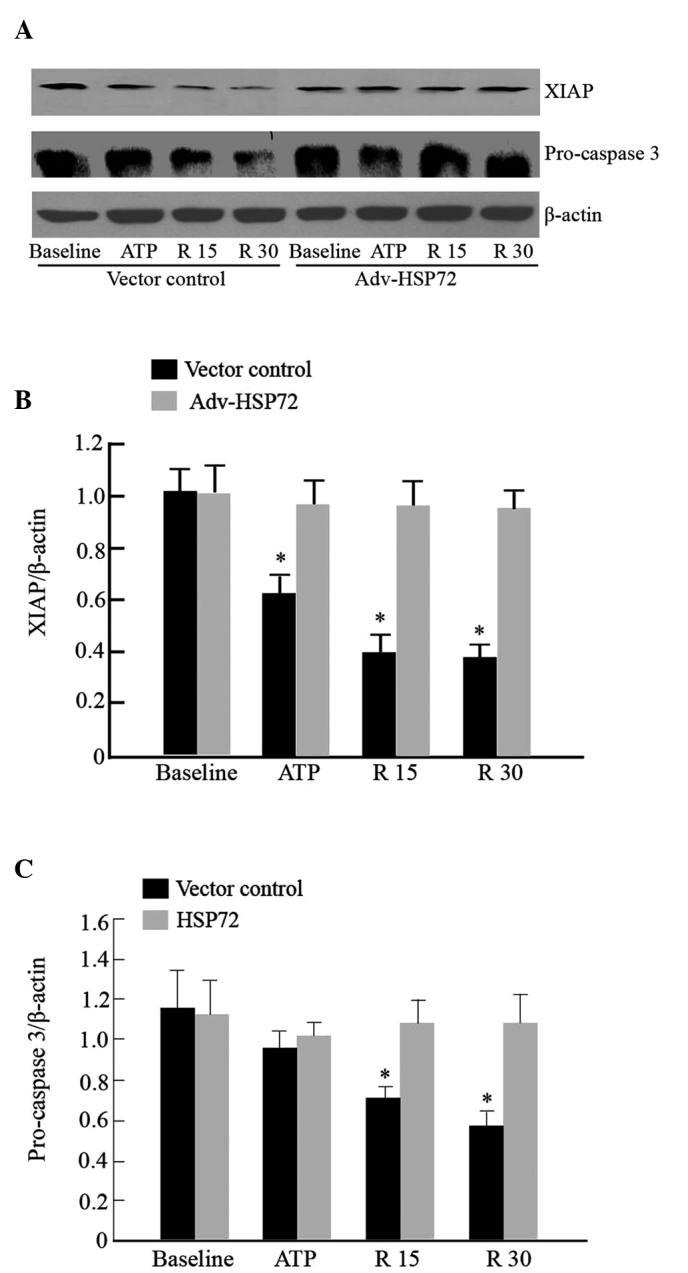
Effect of HSP72 on the expression of apoptotic proteins. (A) XIAP and pro-caspase 3 were assessed by immunoblot following overexpressing HSP72. Empty vector served as negative control; (B) Quantitative determination of the relative abundance of XIAP among different groups. (C) Quantitative determination of the relative abundance of pro-caspase 3 among different groups. Data in B and C are expressed as the mean ± standard error of the mean of three experiments. ^*^P<0.01 versus empty vector control. XIAP, X-linked inhibitor of apoptosis protein; ATP, adenosine triphosphate; HSP72, heat shock protein 72.

**Figure 5:**
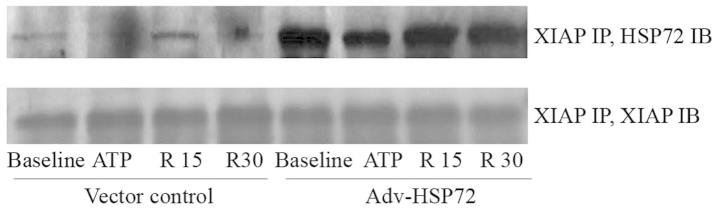
Interaction between HSP72 and XIAP. XIAP was immunoprecipitated from whole Human kidney-2 cell lysates among different groups using a rabbit polyclonal anti-XIAP antibody. HSP72 content was assessed using an anti-HSP72 antibody (upper panel). Immunoblot analysis was used to localize XIAP in cell lysates (lower panel). XIAP, X-linked inhibitor of apoptosis protein; ATP, adenosine triphosphate; IP, immunoprecipitation; IB, immunoblotting; HSP72, heat shock protein 72.

XIAP is degraded during apoptosis induction (21). Since HSP72 preserved XIAP protein content in rats subjected to renal I/R, whether XIAP degradation may be suppressed by HSP72 in HK-2 cells was further examined. Consistent with our *in vivo* findings, overexpression of HSP72 increased the stabilization of XIAP protein following ATP depletion, compared with that in the empty vector control cells (Fig. 4A and B). To examine the possibility that HSP72 may prevent XIAP degradation by binding to XIAP, immunoprecipitation of the two proteins was performed. In the empty vector control cells, interaction between HSP72 and XIAP was weak, whereas overexpression of HSP72 significantly elevated the interaction between these two proteins under all experimental conditions. The immunoprecipitable XIAP content was similar (Fig. 5). The present results suggest that HSP72 may confer XIAP stability against degradation through binding XIAP.

## Discussion

The present study demonstrated that induction of HSP72 by GGA preserved renal XIAP protein content, attenuated tubular cell apoptosis and improved renal dysfunction following I/R injury. *In vitro* experiments in HK-2 cells revealed that overexpression of HSP72 inhibited mitochondrial Smac/DIABLO release, interacted with XIAP and increased levels of XIAP and pro-caspase 3 in ATP-depleted cells. These findings suggest that elevated expression of HSP72 prevents the onset of tubular cell apoptosis in renal I/R injury through suppression of Smac/DIABLO release from mitochondria and restoring XIAP and pro-caspase 3 protein level.

Transient ischemia due to hypovolemia, hypotension or heart failure commonly causes AKI, a disease associated with high mortality, which is increasing in prevalence. Experimental and human studies indicate that the mitochondrial-mediated apoptotic pathway contributes to tubular cell detachment, loss and dysfunction in the course of acute and chronic renal injury (8,22–25). The present study, as well as previous studies (22,24), demonstrated that tubular apoptosis was markedly increased in the I/R-subjected kidney compared with the sham-surgery-subjected kidney. Notable evidence indicates that an alteration in mitochondrial membrane integrity is crucial to the regulation of apoptosis, as stress-mediated permeabilization permits pro-apoptotic factors from the mitochondria into the cytosol and activates caspase-dependent and independent pathways (1,2). The present study demonstrated that mitochondrial Smac/DIABLO, like cytochrome c and AIF (7,15) rapidly release into the cytosol of renal tubular cells during exposure to metabolic inhibitors. During recovery from ATP depletion and following release of mitochondrial Smac/DIABLO, the protein levels of pro-caspase 3 and the caspase inhibitory function of XIAP were reduced. These findings are in agreement with those of previous studies and suggest that Smac/DIABLO from mitochondria ensures continued caspase activation, which is essential ultimately for cell death by inhibiting the caspase inhibitory function of IAPs (3,5,26)

HSP72 is an abundant, inducible molecular chaperone. Multiple studies have demonstrated that HSP72 may protect a variety of cells, including renal tubule cells, against thermal, toxic and ATP depletion-induced damage *in vitro* (7,15,27). Overexpression of HSP72 also suppresses I/R-mediated myocardial, liver, brain and renal injury in animals (28–30). Through release of mitochondrial Smac/DIABLO, it is hypothesized that HSP72 regulates Smac/DIABLO translocation and promotes cell survival during ischemic AKI. In our previous study, administered GGA specifically enhanced the expression of HSP72 in the kidney (16). The present data revealed that pre-conditioning rats with GGA attenuated tubular epithelial cell injury, apoptosis and renal dysfunction. The *in vitro* data substantiated the cytoprotective function of HSP72. As observed following ischemia *in vivo*, overexpressed HSP72 in cultured HK-2 cells attenuated mitochondrial Smac/DIABLO release into the cytosol, degradation of XIAP and the activation of caspase 3. Since HSP72 has been observed to bind to various signaling molecules, it may impact on cell survival. In the present study, the interaction between HSP72 and XIAP was increased under pathophysiological conditions and even elevated following overexpression of HSP72 in the cells. Smac/DIABLO was found to promote caspase activation by binding and neutralizing the IAPs, including XIAP (3). The present observations indicate that HSP72 functions as a regulator that prevents XIAP degradation upon apoptotic signaling, competitively inhibits the binding of XIAP and Smac/DIABLO and maintains the association of XIAP with caspase, thereby preventing apoptosis.

In conclusion, the present study has revealed that HSP72 prevents tubular apoptosis and dysfunction in renal I/R injury by suppressing the release of mitochondrial Smac/DIABLO and protecting the functions of pro-apoptotic proteins.
